# qmotif: determination of telomere content from whole-genome sequence data

**DOI:** 10.1093/bioadv/vbac005

**Published:** 2022-01-31

**Authors:** Oliver Holmes, Katia Nones, Yue Hang Tang, Kelly A Loffler, Michael Lee, Ann-Marie Patch, Rebecca A Dagg, Loretta M S Lau, Conrad Leonard, Scott Wood, Qinying Xu, Hilda A Pickett, Roger R Reddel, Andrew P Barbour, Sean M Grimmond, Nicola Waddell, John V Pearson

**Affiliations:** 1 QIMR Berghofer Medical Research Institute, Herston, Brisbane 4006, QLD, Australia; 2 Institute for Molecular Bioscience, University of Queensland, St Lucia, Brisbane 4072, QLD, Australia; 3 Surgical Oncology Group, Diamantina Institute, The University of Queensland, Translational Research Institute at the Princess Alexandra Hospital, Woolloongabba, Brisbane 4102, QLD, Australia; 4 Flinders Health and Medical Research Institute, Flinders University, Bedford Park, SA 5042, Australia; 5 Children’s Medical Research Institute, Faculty of Medicine and Health, University of Sydney, Westmead, NSW 2145, Australia; 6 Children’s Hospital at Westmead, University of Sydney, Westmead, NSW 2145, Australia; 7 University of Melbourne, Centre for Cancer Research, Victorian Comprehensive Cancer Centre, Melbourne, VIC 3000, Australia

## Abstract

**Motivation:**

Changes in telomere length have been observed in cancer and can be indicative of mechanisms involved in carcinogenesis. Most methods used to estimate telomere length require laboratory analysis of DNA samples. Here, we present qmotif, a fast and easy tool that determines telomeric repeat sequences content as an estimate of telomere length directly from whole-genome sequencing.

**Results:**

qmotif shows similar results to quantitative PCR, the standard method for high-throughput clinical telomere length quantification. qmotif output correlates strongly with the output of other tools for determining telomere sequence content, TelSeq and TelomereHunter, but can run in a fraction of the time—usually under a minute.

**Availability and implementation:**

qmotif is implemented in Java and source code is available at https://github.com/AdamaJava/adamajava, with instructions on how to build and use the application available from https://adamajava.readthedocs.io/en/latest/.

**Supplementary information:**

Supplementary data are available at *Bioinformatics Advances* online.

## 1 Introduction

Telomeres are long-repetitive DNA stretches of (TTAGGG)n repeats at the ends of chromosomes that confer stability, preventing chromosomes from being degraded or fusing to one another. Over time telomeres become shortened as a consequence of cell division which eventually triggers cells to enter senescence and undergo apoptosis. Telomere shortening is a normal part of the ageing process but has also been observed in some diseases including cancer. In cancer, escaping senescence by activation of a mechanism that counteracts telomere shortening is an important requirement for continued proliferation (Hanahan and Weinberg, 2011). Some of the underlying somatic alterations associated with telomere length have been identified in several genes including DAXX, ATRX and TERT (Barthel *et al.*, 2017).

Multiple laboratory-based techniques exist for measuring telomere length, each with its own distinct advantages and disadvantages. Assays that use genomic DNA as the starting material include terminal restriction fragment length analysis, a Southern blot-based technique to separate intact telomere restriction fragments which are then visualized using a telomere repeat probe (Kimura *et al.*, 2010); and single telomere length analysis which exploits the telomeric 3' overhang to amplify individual chromosome-specific telomeres (Baird *et al.*, 2003). However, neither of these techniques is trivial. A widely used telomere length test for molecular, clinical and epidemiological studies is quantitative PCR (qPCR; Cawthon, 2002; O'Callaghan and Fenech, 2011), in which telomeric DNA is amplified and quantitated against amplification of a single-copy gene. Although this technique provides a relative mean telomere length measurement, it can be affected by copy number variation in karyotypically unstable samples. Another DNA-based approach to estimate telomere length is through whole-genome sequencing which is increasingly being used to reveal the mutational landscape and drivers of a variety of cancer types (Hayward *et al.*, 2017; ICGC/TCGA Pan-Cancer Analysis of Whole Genomes Consortium, 2020; Scarpa *et al.*, 2017; Waddell *et al.*, 2015). Whole-genome sequencing allows estimation of telomere length directly from the sequencing data and several approaches have been developed including TelSeq (Ding *et al.*, 2014), Computel (Nersisyan and Arakelyan, 2015) and TelomereHunter (Feuerbach *et al.*, 2019).

Here, we describe qmotif, a fast, efficient (multi-threaded), simple-to-use software package that can quantify telomeric read content from whole-genome sequencing. qmotif has been previously benched marked against other tools by Lee *et al.* (2017) using a panel of cell strains and cell lines with different telomere lengths and was found to outperform other tools tested and had a high correlation with qPCR as well as the shortest run time (Lee *et al.*, 2017). qmotif is written in Java for portability, runs directly against BAM files, has no dependencies on external software, and is faster than existing telomere quantification software.

## 2 Methods

### 2.1 Approach

qmotif is a tool for counting motifs in genome sequencing files and, for increased speed, uses a two-pass matching system. The design intent was that stage 1 be a quick string-equality match and only reads that pass stage 1 go on to a much slower regular expression (regex) match in stage 2. Using a regex in stage 1 on a large BAM significantly increases runtimes and using simple string matching in stage 2 reduces sensitivity leading to missed motif-containing reads. For the purpose of telomere quantification, we typically use a string that represents three concurrent repeats of the canonical telomere motif (TTAGGG) as the stage 1 match and a simple regex for stage 2 which captures any read with two or more concurrent occurrences of the telomeric repeat with variation allowed in the first three bases. Two repeats were selected as the count for stage 1 as it has been shown that this is the threshold at which selecting sequencing reads for telomere analysis maximizes the correlation with laboratory assays (Ding *et al.*, 2014). Every read in the input BAM is put through the stage 1 match and if the read passes the stage 1 match, it goes on to the stage 2 matching which could be against a string or a regex. If the read passes the stage 2 match, the actual matches are retrieved and a tally is kept of the location, count and type of matching motifs.qmotif can be run in a mode where it only processes pre-defined regions of the genome and we use this feature in telomere quantification. Empirical testing has shown that reads containing the telomeric motif, regardless of true genomic location, map to a very small number of locations on our current reference genome assemblies. We can use this prior knowledge to vastly speed up telomere quantification by only processing the pre-defined ‘telomeric’ regions of the genome plus any unmapped reads which captures reads that are telomeric but do not align well to the reference genome.

### 2.2 Required input

qmotif is implemented in Java using the Picard library (version 1.110) and is driven by a single plain-text configuration file in the ‘Windows INI-file’ style. The input is a whole-genome sequencing BAM file that has been duplicate-marked and coordinate-sorted. qmotif is multi-threaded and ∼3 GB of memory are required for each core used. More details on the configuration file and the required inputs can be found on the qmotif wiki page: https://adamajava.readthedocs.io/en/latest/qmotif/index.html.

### 2.3 Test dataset and benchmarking

Here, qmotif was used to analyse whole-genome sequence data to predict the relative telomere length of 21 oesophageal adenocarcinoma tumour/normal pairs. The whole-genome sequence data were previously described (Nones *et al.*, 2014) and are available from the European Genome-phenome Archive (EGAS00001000750). The dataset used in this app note is from oesophageal cancer which is known to have shorter telomeres (Nones *et al.*, 2014). Tumour samples had an average read depth of 76× and the normal had 40×. BAM sizes were on average 215 GB for tumour and 106 GB for normal samples (Supplementary Table S1). The same DNA used for whole-genome underwent qPCR by two independent laboratories using a previously described method (O'Callaghan and Fenech, 2011). qmotif was also compared to Telseq (Ding *et al.*, 2014), one of the most highly cited and commonly used software tool for telomere analysis, and TelomereHunter (Feuerbach *et al.*, 2019) a more recent tool. TelSeq was run using the default parameters which was seven repeats and TelomereHunter was run with three.

## 3 Results

Here, we compared qmotif with qPCR to estimate telomere length from the same DNA extraction used for whole-genome sequencing. We also compare qmotif to two independent tools TelSeq and TelomereHunter using the same whole-genome sequencing dataset.

### 3.1 Qmotif runtime

qmotif can be instructed to look for telomeric repeats at certain regions of the genome that are most likely to contain reads that were aligned to the telomeric areas. This makes the application very fast, qmotif runtimes for the samples in the cohort reported was under 1 min ranging 00:00:17 to 00:00:55, whereas TelSeq (Ding *et al.*, 2014) runtimes ranged from 01:32:13 to 07:10:38 and TelomereHunter (Feuerbach *et al.*, 2019) ranged from 04:26:15 to 19:49:39.

### 3.2 Qmotif compared to laboratory-based assay of telomere length and existing tools

**Fig. 1.**
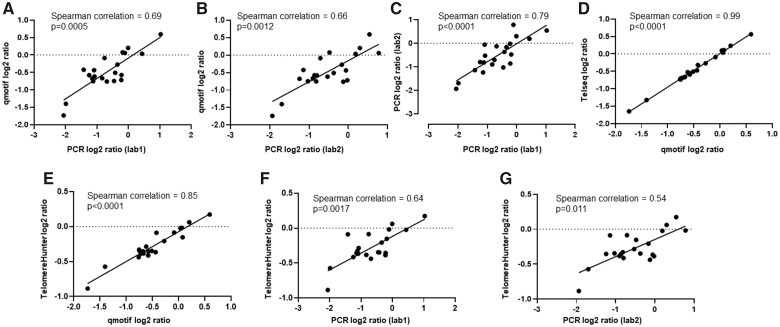
Comparison of qmotif estimates against qPCR performed by two independent laboratories, TelSeq and TelomereHunter. (**A and B**) Plots present log2 of the telomere ratio between tumour/normal as estimated by qmotif (*y*-axis) and qPCR (*x*-axis) from Lab 1 and Lab 2, respectively. (**C**) Correlation of log 2 ratio of tumour/normal telomere length estimated by qPCR by two independent laboratories. (**D**) Correlation of qmotif to TelSeq. (**E**) Correlation of qmotif to TelomereHunter. (**F and G**) Correlation of TelomereHunter and qPCR for two independent labs

Telomere length tumour/normal ratios determined by qmotif were compared to results from qPCR (O'Callaghan and Fenech, 2011) performed by two independent laboratories using the same DNA samples. Spearman correlations between qmotif and the independent qPCR results were 0.69 and 0.66 (Fig. 1A and B), which are similar to the correlation between the two sets of qPCR results (Spearman correlation 0.79; Fig. 1C). This suggests that qmotif analysis of whole-genome sequence data is comparable to qPCR in estimating relative telomere length, as there is similar variability of qPCR estimates between different laboratories.

A comparison of qmotif and TelSeq for the same sequencing data achieved a high correlation (Spearman correlation 0.99; Fig. 1D). A similar correlation was obtained with TelomereHunter (Spearman correlation 0.85; Fig. 1E). Correlations between TelomereHunter and qPCR estimates from two independent labs are presented in Figure 1F and G. The lower correlation of the tools and qPCR observed in this dataset compared to those observed by Lee *et al.* (2017) could be related to the difference in the range of telomere length between the datasets. Here, most tumours have short telomeres.

qmotif is an efficient and accurate way to estimate relative telomere length using short-read whole-genome sequencing data, which requires no extra sample material or laboratory analysis and produces comparable results to laboratory methods. It may be applied to existing and novel whole-genome sequencing datasets, including long-read sequencing, and is faster than other software while returning comparable results thus reducing computational cost.

## Funding

This work was supported by the National Health and Medical Research Council of Australia [NHMRC; grant number APP1021403]. N.W. is supported by an NHMRC fellowship [grant number APP1139071]. This research was performed on QIMR Berghofer computing infrastructure supported by the John Thomas Wilson Endowment and The Ian Potter Foundation.

*Conflict of Interest:* N.W. and J.V.P. are co-founders of genomiQa, equity holders and members of the genomiQa Board.

## Supplementary Material

vbac005_Supplementary_Data
